# The Immune Protection Induced by a Serine Protease Inhibitor From the Foodborne Parasite *Trichinella spiralis*

**DOI:** 10.3389/fmicb.2018.01544

**Published:** 2018-07-11

**Authors:** Yan Y. Song, Yao Zhang, Daqi Yang, Hua N. Ren, Ge G. Sun, Peng Jiang, Ruo D. Liu, Xi Zhang, Jing Cui, Zhong Q. Wang

**Affiliations:** Department of Parasitology, Medical College, Zhengzhou University, Zhengzhou, China

**Keywords:** *Trichinella spiralis*, serine protease inhibitors, identification, tissue localization, immune protection

## Abstract

Serine protease inhibitors (SPI) are a superfamily of the proteins able to suppress serine protease activity, and may exert the major biological function in complement activation, inflammation, and fibrinolysis. A SPI was identified from *Trichinella spiralis* adult worms (AW) by immunoproteomics with early infection sera. The aim of this study was to investigate the protective immune elicited by TsSPI. The complete TsSPI cDNA sequence was cloned into pQE-80 L and then expressed in *Escherichia coli* BL21. The rTsSPI was purified and its antigenicity was determined by Western blotting analysis. By using anti-rTsSPI serum the native TsSPI was identified in somatic and ES proteins from muscle larvae (ML). The results of qPCR and immunofluorescence assay (IFA) revealed that the expression of the TsSPI gene was observed throughout all developmental stages of *T. spiralis* (ML, intestinal infective larvale, 3- and 6-days AW, and newborn larvae, NBL), located principally in cuticles, stichosome, and embryos of this parasitic nematode. Vaccination of mice with rTsSPI triggered high level of anti-TsSPI IgG response, and showed a 62.2 and 57.25% worm burden reduction in the recovery of intestinal AW at 6 days post-infection (dpi) and ML at 35 dpi, respectively. The TsSPI might be a novel potential target for anti-*Trichinella* vaccine.

## Introduction

Trichinellosis, caused by infection with intracellular parasitic nematode *Trichinella* spp., is a major foodborne parasitosis. A total of 65,818 patients of trichinellosis with 42 deaths were recorded from 41 countries during 1986–2009 (Murrell and Pozio, 2011). In a natural environment, more than 150 mammalian animals can be infected with this nematode (Pozio, 2005). Human infection is mainly due to the ingestion of raw or semi-cooked meat infected with *Trichinella spiralis* muscle larvae (ML). In China, pork and pork products are the principal infectious source of human trichinellosis (Cui et al., 2013a; Jiang et al., 2016), 85.71% (12/15) of trichinellosis outbreaks are due to the ingestion of raw or poorly cooked pork during from 2004 to 2009 (Cui and Wang, 2011; Cui et al., 2011). Although mandatory meat inspection is the best strategy for ensuring meat safety, the cost for *Trichinella* inspection by artificial digestion is usually high in small slaughterhouses (Gajadhar et al., 2009; Pozio, 2014). Trichinellosis is an important public health problem; it is an economic hazard on the pork industry and pork food safety (Bai et al., 2017). Accordingly, it needs a reliable method for preventing swine from *Trichinella* infection to ensure meat safety and blocking transmission from swine to human (Yang Y. et al., 2010; Xu et al., 2017b).

After the *Trichinella*-infected meat was ingested by host, the ML are liberated in host’s stomach, migrate to small intestine, subsequently develop into intestinal infective larvae (IIL) 0.9 h post-infection (hpi) (Ren et al., 2011). The IIL penetrate into intestinal mucosa, and develop into adult worms (AW) 31 hpi, the females and males mate and give birth to newborn larvae (NBL), which migrate to skeletal muscles where they finally develop the encapsulated ML (Campbell, 1983). The ML can survive in host’s muscles for 1–2 years even up to 10–15 years without any major harm (Dupouy-Camet et al., 2002; Li et al., 2015), but the mechanism of immune escape of *T. spiralis* ML in hosts is not clear.

Serine protease inhibitors (SPI) are a superfamily of the conserved proteins able to inhibit enzymatic activity of serine proteases and play a major role in complement activation, blood coagulation, inflammation, and fibrinolysis (Gettins, 2002; Molehin et al., 2012). The main characteristic structure of serpin is to have a reactive center loop (RCL), these proteins carry a scissile bond which can be cleaved by serine proteases (van Gent et al., 2003). The serpins released by helminths protect the worms from hydrolytic function of host’s serine proteases, help them to penetrate the defensive barriers, to escape immune attack, and are favorable to the parasite’s survival and colonization in the hosts (Dzik, 2006). Some serpins are identified from parasitic nematodes *Brugia malayi* (Zang et al., 2000), *Ancylostoma caninum* (Duggan et al., 1999), and *Trichuris suis* (Rhoads et al., 2000). A serpin of *Schistosoma mansoni* was highly expressed in the head gland of schistosomules and in adult parasites, which may facilitate the worm survival within dermis and vein (Pakchotanon et al., 2016). A *T. spiralis* serpin from the ML (Uniprot code Q9NH65, GenBank accession no. AF231948) has been identified and expressed. This protein inhibits trypsin activity (Nagano et al., 2001). The protein is possibly related to immune regulation and worm surviving by means of interfering with the immune response of the hosts.

Previous studies showed a serine protease inhibitor of *T. spiralis* (TsSPI) (GenBank accession no. XP_003377380.1) identified in the *T. spiralis* AW excretory/secretory (ES) products by immunoproteomics using early infection sera from patients with trichinellosis (Mitreva et al., 2011; Wang et al., 2017). The aim of the present study was to express the TsSPI in a prokaryotic system and to evaluate its ability to induce immune protection against challenge infection.

## Materials and Methods

### Ethics Statement

This research was performed on the basis of National Guidelines for Experimental Animal Welfare (MOST of People’s Republic of China, 2006). All of the animal experiments were approved by the Zhengzhou University Life Science Ethics Committee (No. SCXK 2015–0005).

### Mice and Worms

Female BALB/c mice, 6 weeks old, were obtained from the Zhengzhou University Experimental Animal Center (Zhengzhou, China). The mice were kept in individual ventilated cages (IVC, Suzhou Fengshi Laboratory Animal Equipment Co., Ltd, Suzhou, China). The strain (ISS534) of *T. spiralis* used in this study was obtained from a domestic pig in central China. This strain was maintained by passage in mice every 6 months.

### Collection of Different Worm Stages and Antigen Preparation

The ML were recovered by the artificial digestion of mouse carcasses infected with *T. spiralis* at 42 days dpi (Gamble et al., 2000; Li et al., 2010). The IIL were obtained from small intestine of the infected mice at 6 hpi (Liu et al., 2016a), and the AW were recovered from intestine at 3 and 6 days pi respectively (Wang et al., 2017). The NBL was obtained from 6 dpi female adults cultured for 24 h at 37°C in RPMI-1640 media (Wu et al., 2016). Soluble crude somatic proteins of IIL, ML, AW, and NBL, and ML ES proteins were obtained as previously described (Wang L. et al., 2013; Yang et al., 2015).

### TsSPI Sequence Analysis

The complete TsSPI cDNA sequence was obtained from GenBank (accession no. XP_003377380.1). Prediction of chemical and physical characteristics including a signal peptide of TsSPI proteins were carried out (Petersen et al., 2011). The transmembrane helices were predicted by Pepstats (Rice et al., 2000). The structural domain of TsSPI was analyzed by means of Simple Modular Architecture Research Tool (SMART) (Sadowski and Jones, 2009). The TsSPI subcellular localization was predicted with TargetP 1.1 Server^1^. The tertiary structure of TsSPI protein was predicted using the PyMOL software. The functional sites of the TsSPI were analyzed by CN3D software.

### Multiple Sequence Alignment and Phylogenetic Analysis

The multiple alignments of sequences with the serine protease inhibitor homologs from other organisms were conducted by using BioEdit Sequence Alignment Editor (Apweiler et al., 2004) and Clustal W (Larkin et al., 2007). The evolutionary relationships of TsSPI to other homologs were assayed with a phylogenetic tree based on a Neighbor-joining (NJ) method analysis with 1,000 bootstrap replications in the MEGA version 5 (Saitou and Nei, 1987). The GenBank accession numbers of each SPI was as follows: *Trichinella spiralis* (XP_0033773 80.1), *Trichinella nativa* (KRZ53349.1), *Trichinella britovi* (KRY55578.1), *Trichinella* sp.T8 (KRZ93426.1), *Trichinella nelsoni* (KRX25675.1), *Trichinella* sp.T9 (KRX62984.1), *Trichinella patagoniensis* (KRY12379.1), *Trichinella* sp.T6 (KRX76834.1), *Trichinella murrelli* (KRX36705.1), *Trichinella pseudospiralis* (KRZ34691.1), *Trichinella zimbabwensis* (KRZ19045.1), *Trichinella papuae* (KRZ74156.1), *Trichuris trichiura* (CDW59461.1), *Trichuris suis* (KFD59516.1), *Pedosphaera parvula* Ellin514 (EEF59283.1), *Pedosphaera parvula* (WP-050785894.1), *Xenopus laevis* (NP-001089382.1), *Xenopus tropicalis* (NP-001011419.1), *Fundulus heteroclitus* (JAR80485.1), *Callithrix jacchus* (JAB48451.1), *Mus musculus* (NP-766639.2), *Homo sapiens* (NP-109591.1). Bootstrap values higher than 60 are shown at branches. The tree was rooted by *Homo sapiens*.

### Cloning, Expression, and Purification of rTsSPI

Extraction of total RNA from *T. spiralis* 3 days AW was carried out by Trizol (Invitrogen, United States) in accordance with the manufacturer’s protocol. The complete TsSPI cDNA sequence was amplified by PCR using specific primers with BamH I and Sac I restriction enzyme sites (shadowed and in italics) (5′-CACCATCACCATCAC*GGATCC*ATGTCGTCCGTCAATTTC GA-3′ and 5′-TCGACCCGGGGTACC*GAGCTC*TTAACCACGATAGCTTCCCA-3′). The PCR amplification reaction contained 25 μl premix (DNA polymerase, dNTPs and PCR buffer), 0.5 μl cDNA, 0.4 μl DNA polymerase, 1.0 μl 10 μM of each primer, 22 μl ddH_2_O. The amplification was performed as follow: 98°C for 5 min; 30 cycles of at 94°C for 3 min, 94°C for 45 s, 60°C for 45 s, and 72°C for 90 s, and finally at 72°C for 5 min. The PCR products were analyzed with 1% agarose gel. The amplified PCR products were purified, digested, and cloned into the pQE-80L containing a N-terminus His-tag (Novagen, Madison, WI, United States). Then, the recombinant pQE-80L/TsSPI was transformed into *Escherichia coli* BL21(DE3) (Madison, WI, United States). The expression of the rTsSPI was induced at 37°C for 5 h by using 0.5 mM IPTG. The rTsSPI was purified with a Ni-NTA His-tag affinity kit (Novagen). SDS–PAGE analysis of rTsSPI was executed with at 120 V for 2 h 12% acrylamide separating gel (Wang et al., 2011). The concentration of the rTsSPI was measured (Bradford, 1976).

### Immunization Scheme

Three groups of mice (20 mice per group) were used in this study. Mice were subcutaneously immunized in different site with 20 μg of rTsSPI emulsified with complete Freund’s adjuvant, and boosted three times with the rTsSPI with incomplete Freund’s adjuvant at 2 weeks interval. Control groups were given incomplete Freund’s adjuvant alone or PBS at the same time intervals as the experimental groups (Cui et al., 2013b; Gu et al., 2017). Hundred microliter of blood were collected from the tail of each mouse before immunization and then at 2, 4, 6, and 8 weeks after immunization. Individual serum samples were kept at –40°C until used.

### ELISA for Detection of rTsSPI-Specific Antibodies

Antibodies against the rTsSP1 (total IgG, it’s subtypes IgG1 and IgG2a) in all vaccinated mice were determined by ELISA at 2 weeks after each immunization (Long et al., 2014; Liu et al., 2017). ELISA plates were coated with rTsSPI (1.5 μg/mL) by incubation at 37°C for 2 h. After washing with PBS-0.5% Tween 20 (PBST) plates were blocked with 200 μL of PBST-5% skim milk. Mouse serum samples at 1:100 dilution were then added and plates were incubated at 37°C for 1 h. Goat anti-mouse IgG (IgG1 or IgG2a)-HRP conjugate at 1:5,000 dilution (Sigma, United States) was used as the secondary antibodies. Reaction was allowed to develop by the addition o-phenylenediamine dihydrochloride substrate (OPD; Sigma) as well as 30% H_2_O_2_ for 30 min, and the reaction was stopped by adding 2 M H_2_SO_4_. A microplate reader (Tecan, Schweiz, Switzerland) was used to determine the absorbance at 492 nm (Gu et al., 2017; Liu et al., 2017).

### SDS–PAGE and Western Blotting Analysis of rTsSPI

Protein samples for SDS–PAGE analysis were loaded in each lane as follows: 5 μg rTsSPI, 12 μg ML somatic proteins and ML ES proteins per lane. SDS–PAGE was carried out using 12% polyacrylamide gels; the gel was subsequently transferred for 35 min at 18 V onto the membranes in a semi-dry transfer cell (Bio-Rad, United States) (Liu P. et al., 2015). After being blotted, the membranes were blocked at 37°C for 1 h with TBST-5% skim milk, and incubated at 37°C for 1 h with 1:100 dilutions of mouse serum samples (infection serum, pre-immune serum and anti-rTsSPI serum). The membranes were washed, and incubated at 37°C for 1 h with goat anti-mouse IgG-HRP conjugate (1:5,000 dilution; Sigma-Aldrich). Finally, the membranes were treated using a substrate 3, 3′-diaminobenzidine tetrahydrochloride (Sigma-Aldrich) (Liu et al., 2016b).

### Quantitative Real-Time PCR (qPCR)

Total RNA of different *T. spiralis* stages (AW, NBL, ML, and IIL) was isolated with Trizol reagent (Invitrogen, Carlsbad, CA, United States). The TsSPI transcriptional level from the different stages was assayed by qPCR as previously described (Long et al., 2015). The specific primers of qPCR for amplifying the TsSPI gene were 5′-TCCAACGTCTTCTTCTCGCC-3′, and 5′-ACAGACTGAACAGGCGATCC-3′. Glyceraldehyde-3-phosphate dehydrogenase of *T. spiralis* (GAPDH, GenBank accession No. AF452239), which was confirmed to be expressed stably and constitutively in different *T. spiralis* lifecycle stages (ML, IIL, AW, and NBL), was utilized as internal control (Nagano et al., 2011; Sun et al., 2018). The data were analyzed according to the comparative Ct (2^-ΔΔ*C*_t_^) method (Liu et al., 2013).

### Indirect Immunofluorescent Assay (IFA)

The expression and localization of the native TsSPI in this nematode were analyzed by IFA using anti-rTsSPI serum (Cui et al., 2015b). The intact worms of various *T. spiralis* stages (AW, NBL, ML, and IIL) were used to examine the TsSPI expression on the surface of the parasite, the localization of the TsSPI in the worm tissues was carried out using 3-μm sections of ML, IIL, and AW fixed in 4% formaldehyde. The intact nematode and its sections were blocked for 1 h at 37°C with 5% goat sera, and then incubated with a 1:10 dilution of mouse serum samples (infection serum, pre-immune serum and anti-rTsSPI serum) at 37°C for 1 h in a humid chamber. After washing with PBST, the worm preparations were incubated for 1 h at 37°C with 1:100 dilutions of an anti-mouse IgG-FITC conjugate (Santa Cruz Biotechnology, Dallas, TX, United States). The washes were repeated, and the tissue sections were observed by fluorescence microscopy (Olympus, Tokyo, Japan) (Zhang et al., 2013).

### Challenge Infection Experiment and Immune Protection Evaluation

Two weeks after the final immunization, each mouse was challenged orally with 300 ML of *T. spiralis*. Ten mice from each group were euthanized at 6 dpi, and intestinal AW were collected and counted (McGuire et al., 2002; Cui et al., 2013b). For detection of ML mice were sacrificed and the number of ML determined at 35 dpi by the artificial digestion of mouse carcasses (Li et al., 2010; Liu P. et al., 2015). In aspects of immune response, serum specific anti-rTsSPI antibodies (total IgG, IgG1, and IgG2a) in all vaccinated mice were measured with ELISA following each vaccination. The immune protection efficacy was evaluated according to worm burden reduction of intestinal adults and larvae per gram (LPG) of muscles obtained from the immunized mice with rTsSPI relative to those of alone PBS control mice (Yang Y. et al., 2010; Xu et al., 2017a).

### Statistical Analysis

The statistical analysis of data was conducted via SPSS 17.0 software. Quantitative data were shown as means ± standard deviation (SD). The comparison of the TsSPI expression level in different *T. spiralis* stages was performed with one-way ANOVA. One-way ANOVA or Student’s *t*-test was used to analyze the differences of intra- and intergroup. Difference was regarded as statistically significant at *P* < 0.05.

## Results

### Bioinformatics Analysis of TsSPI Gene Sequences

**FIGURE 1:**
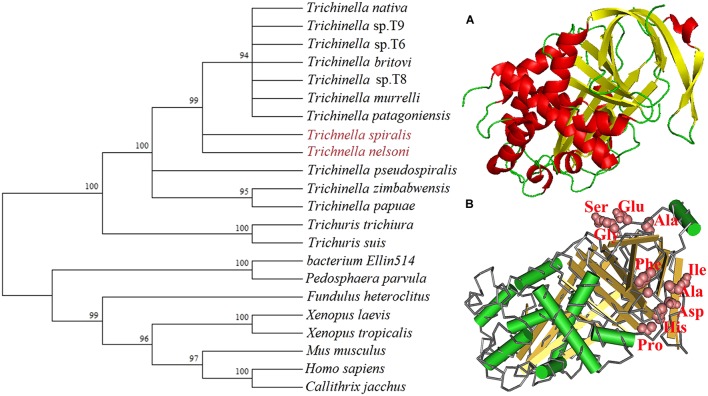
Phylogenetic trees of serine protease inhibitors (SPI) of 22 organisms with the NJ method and mapped with MEGA (left) and the predicted three-dimensional structure of *T. spiralis* TsSPI protein (right). **(A)** The predicted three-dimensional structure of TsSPI protein contains 9 α-helixes (in red) and 12 β-strand (in yellow). **(B)** The functional domain with an active site carrying a classic SPI reactive central loop (RCL) consisted of ten amino acids. The active site of TsSPI is marked on pink.

Bioinformatics analysis showed that the complete TsSPI cDNA sequence was 1,050 bp encoding a protein of 349 amino acids, with a molecular weight (MW) of 39.6 kDa and an isoelectric point (pI) of 5.78. The prediction results of Signal P 4.1 and TMHMM Server revealed that the TsSPI protein had no signal peptides, but it had transmembrane domain, located outside the membrane. The subcellular localization analysis suggested that the peptide chain was located in the mitochondria, periplasm and cytoplasm with the possibility of 16.9, 24.9, and 62%, respectively. The maximum location was in the cytoplasm. The homology comparison between *T. spiralis* and *T. nelsoni* with TsSPI revealed 84% identity at the amino acid level. The phylogenetic analysis of TsSPI relative to the SPI of other organisms was shown in **Figure 1**. The phylogenetic tree constructed with NJ method supported the monophyly of various species of the genus *Trichinella*, and *T. spiralis* has the closest evolutionary relationship with *T. nelsoni*, and is more closely related to serpin of nematodes than those of other organisms.

The SMART analysis showed that the TsSPI protein had 9 α-helixes and 12 β-strand, contained a functional domain (between positions 15 and 349) with an active site carrying the classic reactive central loop (RCL) of SPI, which consisted of ten amino acids (aa 298–301 and aa 323–328) (**Figure 1**).

### Expression and Identification of Recombinant TsSPI Protein

**FIGURE 2:**
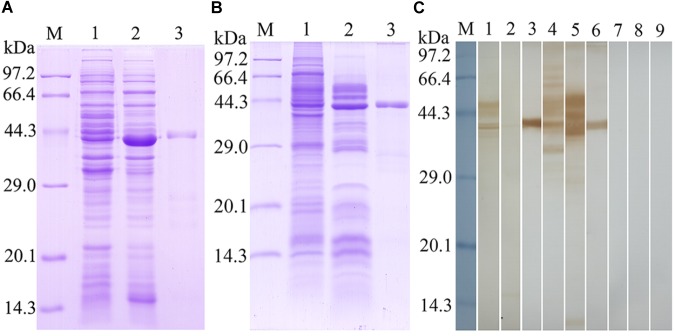
Identification of rTsSPI. **(A)** SDS–PAGE analysis of the rTsSPI. Lane M: molecular weight markers; Lane 1: recombinant bacteria lysate before induction; Lane 2: recombinant bacteria lysate after IPTG induction; Lane 3: the purified rTSPI. **(B)** SDS–PAGE of somatic proteins (Lane 1), ES proteins (Lane 2), and rTspGST (Lane 3). **(C)** Western blotting. The native TsSPI protein in ML somatic proteins (lane 1) and ES proteins (lane 2) as well as the rTsSPI (lane 3) were identified by anti-rTsSPI serum. The ML somatic proteins (lane 4), ES proteins (lane 5) and rTsSPI (lane 6) were also probed with serum samples of *T. spiralis*-infected mice. The ML somatic proteins (lane 7), ES proteins (lane 8), and rTsSPI (lane 9) were not recognized with pre-immune sera of normal mice.

The TsSPI was successfully cloned into prokaryotic expression vector pQE-80L. The rTsSPI was expressed in *E. coli* following induction with IPTG. By SDS–PAGE analysis, the rTsSPI had a MW size of 42.5 kDa after purification and was consistent with its predicted MW (**Figure 2**). On Western blotting analysis, the rTsSPI was detected using anti-rTsSPI serum, but not by pre-immune serum. By using anti-rTsSPI serum, the native TsSPI with 42.5 kDa was identified in *T. spiralis* ML ES proteins, but several native TsSPI proteins (40.7, 42.5, 44.1, and 49.3 kDa) were identified in ML crude somatic proteins (**Figure 2**), indicating that the TsSPI is an excretory/secretory and present in the somatic and ES proteins of the parasite.

### qPCR Analysis of TsSPI Transcription Level at Various Phases

**FIGURE 3:**
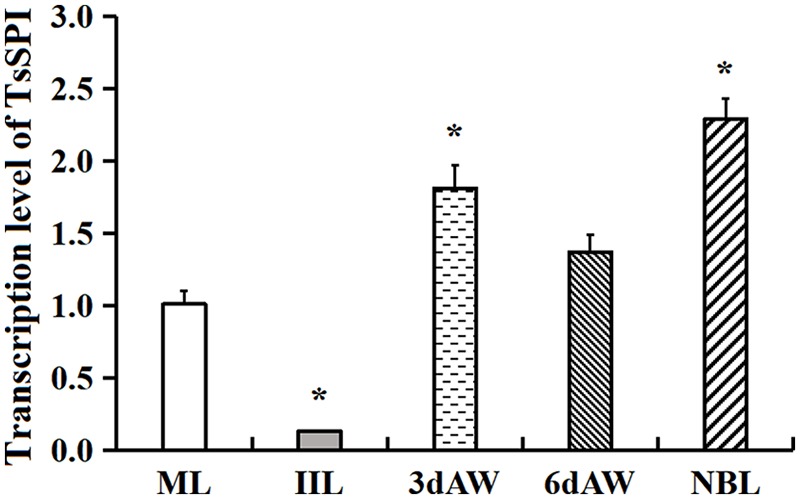
qPCR analysis of TsSPI transcription level at various stages of *T. spiralis*. The TsSPI mRNAs from ML, IIL, 3- and 6-days AW, and NBL were extracted and amplified by qPCR. The TsSPI transcription level was calculated according to the Ct (2^-ΔΔ*C*_t_^) method. The fold change in the TsSPI genes was normalized to G3PDH used as a housekeeping gene control. The data shown are representative from three independent experiment. Statistical differences are marked with asterisks (^∗^) compared with the ML stage at *P* < 0.05.

The transcription level of the TsSPI in different stages of *T. spiralis* was analyzed by qPCR. The qPCR results revealed that the transcription of the TsSPI mRNA was detected through all developmental phases (ML, IIL, 3- and 6-days AW, and NBL) (**Figure 3**). Compared with ML, the TsSPI transcription level in IIL stage was the lowest (-7.77 fold, *F* = 76.00, *P* < 0.01); whereas it was highest in NBL stage (2.27-fold, *F* = 51.13, *P* < 0.05). There are also statistically difference of TsSPI transcription level between ML and 3 days AW stage (1.79 fold, *F* = 15.38 *P* < 0.05), but no difference between ML and 6 days AW (F6d *AW* = 4.61, *P* > 0.05).

### Expression and Tissue Localization of TsSPI at *T. spiralis* by IFA

**FIGURE 4:**
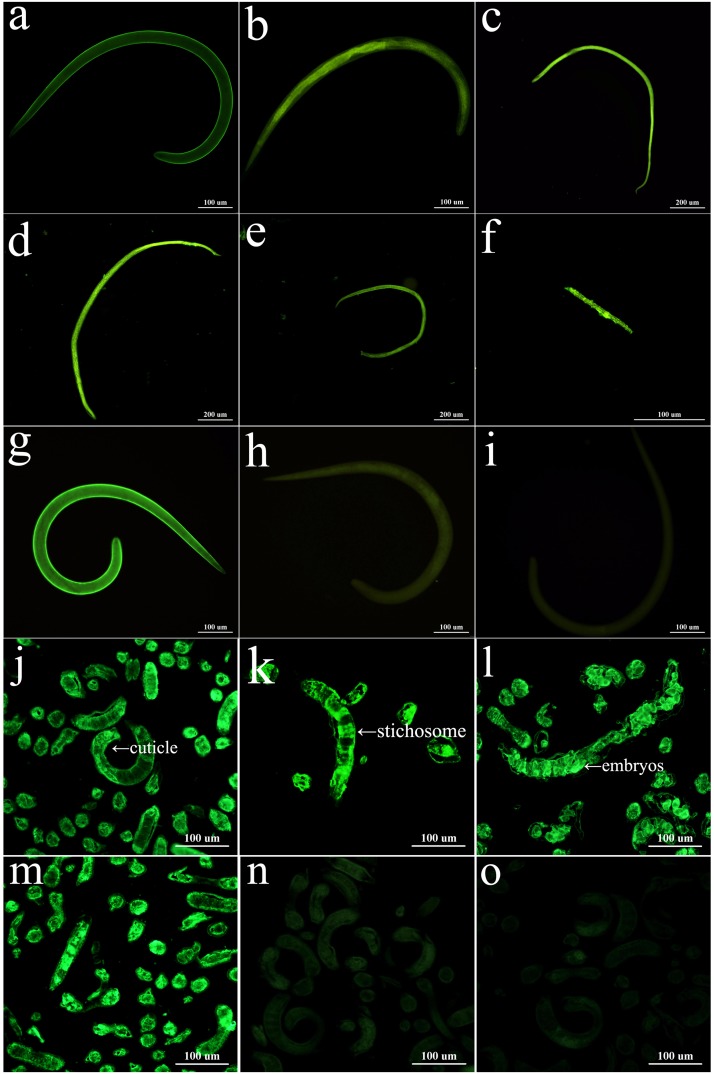
Expression and immunolocalization of TsSPI in different *T. spiralis* phases. **(a–i)** The intact worms were examined by IFA with different mouse sera. When the anti-rTsSPI serum was used, immunostaining was observed on the surface of ML **(a)**, IIL **(b)**, 3-day female adult **(c)**, 6-day female **(d)**, 6-day male adult **(e)**, and NBL **(f)**. ML recognized by infection serum **(g)** was used as a positive serum control; ML incubated using pre-immune serum **(h)** and PBS **(i)** were used as negative serum and blank controls. **(j–o)**: Worm sections were examined by IFA with different mouse sera. The worm sections were probed by anti-rTsSPI serum, intense fluorescent staining is observed in cuticles, stichosome of ML **(j)**, IIL **(k)**, and embryos of 3-day female adult **(i)**. The ML reacted by infection serum **(m)** was used as a positive serum control; there are no staining in the ML with pre-immune serum **(n)** and PBS **(o)** as a negative control. Scale-bars: 100 μm.

IFA with intact worms were used to analyse the expression and tissue localization of TsSPI at various stages of this nematode. By using anti-rTsSPI serum, green fluorescent staining was detected on the surface of various stages (ML, IIL, 3-and 6-day adults, and NBL) with intense staining on the surface of AW and NBL. When the worm sections were probed by anti-rTsSPI serum, the staining was located at the cuticle, stichosome of ML, IIL, and embryos in female adult uterus (**Figure 4**). No immunostaining could be detected at whole larvae and their tissue sections that were probed with pre-immune normal sera.

### Antibody Responses Triggered by Immunization With rTsSPI

**FIGURE 5:**
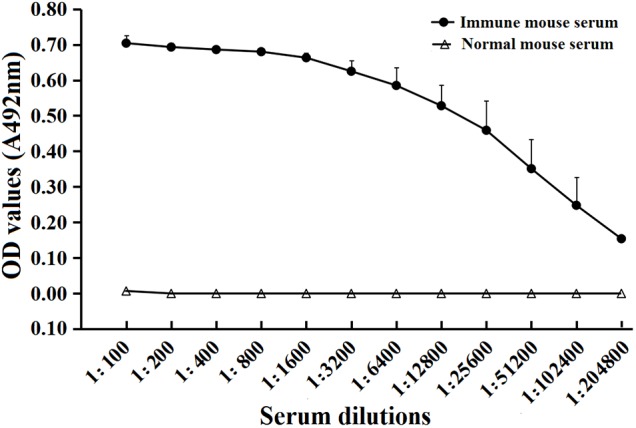
Serum anti-rTsSPI IgG titers after the fourth immunization in immunized mice determined by ELISA. The data are presented as the mean OD values ± SD of the antibody levels from twenty mice.

To determine humoral antibody responses to rTsSPI in immunized mice, serum specific anti-rTsSPI IgG titers at 2 weeks after the fourth immunization were assayed by ELISA with rTsSPI as coating antigen. The results revealed that anti-rTsSPI antibodies were elicited by the immunization with rTsSPI. Serum anti-rTsSPI IgG titer was 1:10,2400 following the final immunization (**Figure 5**), demonstrating that the rTsSPI was highly immunogenic.

**FIGURE 6:**
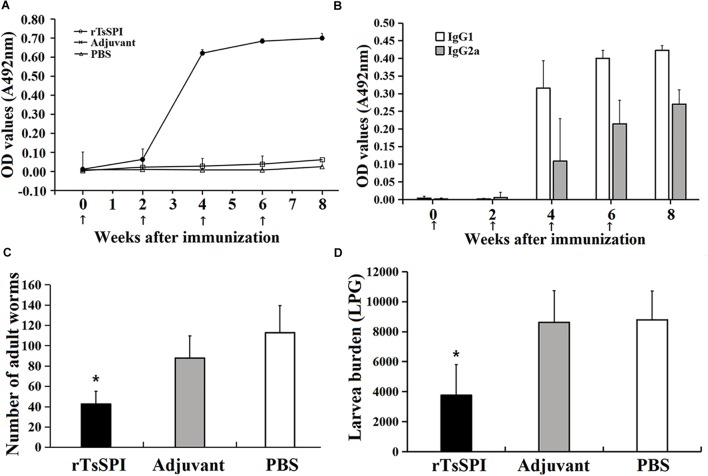
Protective immunity in mice immunized with rTsSPI. Serum IgG **(A)** and IgG subtype **(B)** responses to the rTsSPI at different times post immunization were measured by ELISA. The data are presented as the mean OD values ± SD of specific anti-rTsSPI antibodies from ten mice. The arrow (↑) represents immunization times. The number of adults recovered from intestines **(C)** and larvae per gram (LPG) of muscles **(D)** in immunized mice after challenge with 300 *T. spiralis* muscle larvae. Results are expressed as the mean ± SD of 10 mice per group. An obvious reduction was observed in the worm burdens of intestinal adults at 6 dpi and muscle larvae at 35 dpi (^∗^*P* < 0.01).

Serum level of specific anti-TsSPI IgG, IgG1 as well as IgG2a 2 weeks after each immunization was determined by ELISA. Anti-rTsSPI IgG level was evidently increased after the second boost and peaked at 2 weeks after the third immunization. Nevertheless, the mice injected with alone adjuvant or PBS did not show evident detectable anti-rTsSPI antibody response (**Figure 6A**). Anti-rTsSPI IgG subclass assay revealed that the IgG1 level on weeks 4, 6, and 8 after vaccination was distinctly higher than IgG2a level (*t*_4W_ = 4.713, *t*_6w_ = 7.8, *t*_8W_ = 11.335, *P* < 0.01; **Figure 6B**), demonstrating that the predominant IgG subclass triggered with rTsSPI was IgG1, but the IgG2a antibody response was also induced after the second immunization.

### Immune Protective Efficacy of rTsSPI

Immune protective efficacy of rTsSPI against challenge infection with *T. spiralis* ML was investigated in immunized mice. The mice immunized with rTsSPI exhibited a 62.2% worm burden reduction of intestinal adults at 6 dpi and a 57.25% reduction of ML at 35 dpi (**Figures 6C,D**), in comparison with those of alone PBS control group (F_adult_ = 62.34, F_larvae_ = 27.2, *P* < 0.01). Furthermore, the intestinal adult and muscle larval burden of immunized group was also apparently lower than those of adjuvant-injected group (F_adult_ = 35.77, *P* < 0.01; F_larvae_ = 28.70, *P* < 0.01). However, there were no significantly difference in adult (F_adult_ = 4.43, *P* > 0.05) and larvae (F_adult_ = 0.0327, *P* > 0.05) burden between animals that received adjuvant or PBS.

## Discussion

The TsSPI from *T. spiralis* adult worm was cloned and expressed in the present study. SMART analysis revealed that the TsSPI had a functional domain with an active site containing the characteristic classic serpin features such as the RCL (Molehin et al., 2012). Additionally, the TsSPI protein is composed of 349 aa with a predicted MW of 39.6 kDa and a native MW of 42.5 kDa, which is consistent with other members of the serpin superfamily (Molehin et al., 2014). After purification, the rTsSPI was strongly immunogenic and used for preparing anti-rTsSPI serum. Western blotting revealed that the rTsSPI was detected by anti-rTsSPI serum and infection serum. In addition, the native TsSPI proteins were identified by anti-rTsSPI serum in somatic and ES proteins from *T. spiralis* ML, suggesting TsSPI is one component of somatic and ES proteins of this parasitic nematode. Several native TsSPI with 40.7–49.3 kDa in somatic proteins were recognized by anti-rTsSPI serum, maybe due to the fact that the TsSPI may have different isoforms, or the TsSPI is likely processed via post-translational modifications/alternative splicing (Bien et al., 2015; Cui et al., 2015b; Yang et al., 2017).

The data of qPCR analysis showed that TsSPI transcription was detected throughout all developmental phases of the nematode lifecycle, which were consistent with the fact that the TsSPI gene was identified from *T. spiralis* AW (Wang et al., 2017) and the 3-day AW had contained the NBL embryos (Campbell, 1983). The results of IFA with anti-rTsSPI serum indicated that TsSPI was localized on the surface, cuticle, stichosome, and embryos at all the lifecycle stages of this nematode. *T. spiralis* ES proteins come from mainly the shed or secretory proteins of cuticles and the secretory granules of stichosome (Bioreau et al., 1997; Wang et al., 2014; Liu R.D. et al., 2015), whereas the TsSPI was principally located in cuticle and stichosome of this parasite, so the TsSPI is present in the ES proteins. Although the TsSPI was expressed in all lifecycle stages of the parasite, but the higher expression was found in AW and NBL stages. The TsSPI may play an important role in larval surviving in the process of early development in host. Moreover, our IFA results showed that the TsSPI was widely distributed on the cuticle surface of AW and NBL, suggesting that the TsSPI could protect the worms against digestive attack of proteolytic enzymes from host. The complex of *Trichinella* serpin-host serine protease could also cover the outside surface of *Trichinella* larval cuticles and assist the larval immune escape (Martzen et al., 1985).

Previous studies showed that one recombinant *Brugia malayi* serpin was strongly immunogenic and recognized by immune animal sera (Yenbutr and Scott, 1995). The serom *S. haematobium* was located at the surface of this schistosome and can interact with host’s proteases and cells (Blanton et al., 1994). A serpin from *Schistosoma japonicum* is a tegumental protein; it’s expression is observed merely at adult and cercarial stage, and immunization of mice with the rSj serpin elicited high levels of specific antibodies and produced some protection against challenge infection as indicated by a 36 and 39% reduction of AW and eggs, respectively (Yan et al., 2005). A serpin named AduTIL-1 from hookworm *Ancylostoma duodenale* was cloned and expressed, it had inhibitory activities against human neutrophil elastase and pancreatic trypsin, localized in esophagus, intestine, and cuticle surface of AW. AduTIL-1 may be related with the *Ancylostoma* survival in host by targeting related digestive enzymes and neutrophil elastase (Jin et al., 2011). Two serpins from *T. spiralis* inhibited the activities of chymotrypsin and pepsin (Zhang et al., 2016). Another *T. spiralis* serpin (DQ864973) was detected by screening ML cDNA library with *Trichinella*-infected swine serum, and the rTs-serpin can induce partial protection against *Trichinella* larval challenge in immunized mice as demonstrated by a 33.36% worm burden reduction of intestinal AW at 8 dpi and a 44.82% reduction of ML at 42 dpi, respectively (Xu et al., 2017b). A serpin from *T. pseudospiralis* (GenBank accession no. JF764789.1) have an important function for immunoregulating *Trichinella* infection through activating the M2-polarized signaling pathway (Xu et al., 2017c). Compared with the other *Trichinella* antigens, the TsSPI as a vaccine molecule might have the following advantages: the TsSPI is highly expressed in intestinal stage worms and widely distributed on the cuticle of the nematode, TsSPI could protect intestinal worms from host’s serine proteolysis; whereas high levels of anti-TsSPI antibody produced by rTsSPI immunization could neutralize the anti-proteolytic activity of the TsSPI and inhibit the parasite immune evasion (Yan et al., 2005). Additionally, the formation of TsSPI and anti-TsSPI antibody immune complex may physically block the worm invasion of intestinal mucosa (McVay et al., 2000). Thus, *Trichinella* serpins could be proposed as potential vaccine candidate targets against *Trichinella* infections.

The mice immunized with the rTsSPI exhibited specific Th2-predominant immune response against rTsSPI. The worm burden reduction observed in our study is similar to those in mice immunized with recombinant nudix hydrolase and glutathione S-transferase of *T. spiralis* (Cui et al., 2013b; Long et al., 2014; Liu et al., 2017). The immune protection may be due to the generation of high titer of specific anti-TsSPI IgG, which neutralized the anti-proteolytic activity of the serpin (Yan et al., 2005). Anti-*Trichinella* IgG could bind to the surface proteins of *T. spiralis* larvae and form the immune complex in the larval cephalic portion, which may physically block larval invasion or interfere with larval sensory reception, therefore protect intestinal mucosa against worm invasion (McVay et al., 1998, 2000; Wang B. et al., 2013). It was also reported that anti-*Trichinella* antibodies could kill *T. spiralis* NBL by an ADCC-mediated mechanism with antibody dose-dependent mode (Moskwa, 1999; Cui et al., 2015a; Liu et al., 2018).

To eliminate parasites from the intestines, the protective immune response induced by vaccination should disable, degrade and dislodge the parasites (Maizels et al., 2012). Since *Trichinella* infection is acquired by ingestion of the infected meat, oral or intranasal immunization with rTsSPI protein or DNA vaccine will be more appropriate to induce lasting intestinal protective immune responses to the intestinal *Trichinella* stages (Castillo Alvarez et al., 2013; Pompa-Mera et al., 2014; Liu P. et al., 2015). The mice immunized orally with *Salmonella*-delivered *T. spiralis* paramyosin (TsPmy) DNA vaccine produced a 44.8% reduction of AW and a 46.6% reduction of ML in vaccine mice compared with the PBS control (Wang et al., 2016). Intranasal immunization of mice with attenuated *Salmonella* expressing a *T. spiralis* gp43 antigen-derived 30-mer peptide fused to the molecular adjuvant C3d-P28 produced a 92.8% reduction of intestinal adult worm burden following challenge (Pompa-Mera et al., 2014). Furthermore, mucosal vaccination may provide a needle-free delivery approach, thus increasing safety, accessibility and cost-effectiveness. Therefore, oral or intranasal vaccination with *Trichinella* vaccines can produce the effective local and systematic intestinal mucosal immune responses (Ortega-Pierres et al., 2015).

Additionally, because of the side effects of complete Freund’s adjuvant, the other adjuvants (e.g., methacrylic acid copolymers, MontanideTM IMS1312 and MontanideTM ISA720) should be used to increase the level of immune protection in the TsSPI-immunized animals (Ortega-Pierres et al., 2015). When the adjuvant ISA70 and 720 (W/O emulsion) was used, an obvious decrease in muscle larval burden was observed, whereas animals treated with alum and IMS1312 and 1,313 had a higher larval burden, indicating that the application of ISA70 might enhance the protection against *Trichinella* infection (Deville et al., 2005). The vaccination of mice with rTs-Pmy formulated with either ISA206 or ISA720 induced Th1 and Th2 immune responses similar to those triggered by Freund’s adjuvant formulation and also exhibited a similar level of immune protection against *Trichinella* challenge infection, indicating Montanide ISA206 or ISA720 may be used as an effective and safety vaccine adjuvant (Yang J. et al., 2010). The immunization of mice with rTs-serpin and IMS1313 showed higher humoral immunity and similar levels of cellular immunity and worm reduction rate, when compared with Freund’s complete adjuvant/Freund’s incomplete adjuvant (FCA/FIA) formulated vaccination, suggesting that Montanide IMS 1313 are as effective as FCA but less toxic. The higher humoral immunity induced by rTs-serpin and IMS1313 might be related with the fact that the adjuvant IMS 1313 consisted of water-dispersed liquid nanoparticles combined with an immunostimulating compound enhanced antigen-specific immune responses (Xu et al., 2017b). The enhanced immune protection induced by the adjuvant Montanide IMS might be related with production of higher levels of specific IgG, IgM and IgE antibodies against *T. spiralis* (Deville et al., 2005; Xu et al., 2017b).

Our results indicated that vaccination of mice with TsSPI produced a partial immune protection against *Trichinella* larval challenge; the TsSPI has a potential role as a novel candidate target for vaccine against trichinellosis, but further research is required to identify the biological function and possible immune modulating roles of this serpin in host. The determination of physiological and biological functions of TsSPI will be valuable for understanding of *Trichinella*-host interaction, which might provide new measures for prevention and treatment of trichinellosis.

## Conclusions

The TsSPI was expressed throughout all developmental phases in *T. spiralis* life-cycle, and located principally in cuticle, stichosome and embryos of this foodborne nematode. The immunization of mice with rTsSPI triggered high level of anti-TsSPI IgG responses and produced a partial immune protection against challenge with *T. spiralis* in mice. The TsSPI might be considered as a novel candidate target for vaccine against trichinellosis, but its physiological and biological functions need to be further investigated.

## Author Contributions

JC and ZW designed this study. YS, YZ, DY, HR, GS, RL, PJ, and XZ conducted the experiments. YS, JC, and ZW drafted and revised the manuscript. All authors agreed on the final manuscript to be published.

## Conflict of Interest Statement

The authors declare that the research was conducted in the absence of any commercial or financial relationships that could be construed as a potential conflict of interest.

## Footnotes

1 http://www.cbs.dtu.dk/services/TargetP/
